# Video assisted lobectomy learning curve – what is the magic number?

**DOI:** 10.1186/1749-8090-8-S1-O221

**Published:** 2013-09-11

**Authors:** A Brunswicker, M Berman, M Van Leuven, F Van Tornout, WR Bartosik

**Affiliations:** 1 Thoracic Surgery Department, Norfolk and Norwich University Hospital, Norwich, UK

## Background

Previous publications suggest that surgeons have to overcome a learning curve of 50 cases to be proficient and safe in performing VATS lobectomy.

## Methods

A prospective study of the first 40 consecutive VATS lobectomies performed by a single experienced thoracic surgeon over a 3-year period. All patients were divided equally into two groups with Group A being the first 20 patients. Mortality, operative time, complications, conversion rate and need for blood transfusion were recorded for all patients. All procedures were performed via anterior approach with 5-8 cm utility port and 2 additional 10 mm ports without using ribs spreaders.

## Results

Patients’ median age was 68 (Group A) and 68.5 (Group B). Indications included lung cancer (34), bronchiectasis (2), typical carcinoid (1), benign lesion (1), tuberculosis (1) and leiomyoma (1). There was no mortality or re-do thoracotomy in both groups. None of the patients required a blood transfusion perioperatively. The conversion rate was 25% in group A and 5% in group B. Reasons for conversion were adhesions with bulky N1 disease (4) and undetermined resection margin at frozen section (1). The median operative time was significantly shorter for group B 150 min vs. 192.5 min, p 0.006. Complication rates were equal in both groups at 15%, prolonged air leak (3), atrial fibrillation (2) and bowel obstruction (1). With increasing experience, the overall clinical practice changed: Initially, 25% of all lobectomies were performed thoracoscopically, which has increased to 75% currently.

## Conclusions

The VATS lobectomy learning curve is multifactorial and its steepness seems to be positively influenced by the surgeon’s experience. We have shown that the learning curve for an experienced surgeon can be as short as 20 cases. Senior surgeons, who are willing to learn VATS lobectomy can acquire this technique with as little as 20 cases. However, good institutional support and patient selection are crucial. and patient selection are crucial.

